# Thrombosis Complications in Pediatric Acute Lymphoblastic Leukemia: Risk Factors, Management, and Prevention: Is There Any Role for Pharmacologic Prophylaxis?

**DOI:** 10.3389/fped.2022.828702

**Published:** 2022-03-10

**Authors:** Vilmarie Rodriguez

**Affiliations:** Nationwide Children's Hospital, Columbus, OH, United States

**Keywords:** thrombosis, anticoagulation, acute lymphoblastic leukemia, risk factors, outcomes

## Abstract

Pediatric acute lymphoblastic leukemia (ALL) has achieved close to 90% cure rates through extensive collaborative and integrative molecular research, clinical studies, and advances in supportive care. Despite this high achievement, venous thromboembolic complications (VTE) remain one of the most common and potentially preventable therapy-associated adverse events in ALL. The majority of thromboses events involve the upper central venous system which is related to the use and location of central venous catheters (CVC). The reported rates of symptomatic and asymptomatic CVC-related VTE range from 2.6 to 36.7% and 5.9 to 43%, respectively. Thrombosis can negatively impact not only disease-free survival [e.g., therapy delays and/or interruption, omission of chemotherapy agents (e.g., asparaginase therapy)] but also can result in long-term adverse effects that can impair the quality of life of ALL survivors (e.g., post-thrombotic syndrome, central nervous system (CNS)-thrombosis related complications: seizures, neurocognitive deficits). In this review, will discuss thrombosis pathophysiology in pediatric ALL, risk factors, treatment, and prevention strategies. In addition, the recently published clinical efficacy and safety of direct oral anticoagulants (DOACs) use in thrombosis treatment, and their potential role in primary/secondary thrombosis prevention in pediatric patients with ALL will be discussed. Future clinical trials involving the use of these novel oral anticoagulants should be studied in ALL not only for primary thrombosis prevention but also in the treatment of thrombosis and its secondary prevention. These future research findings could potentially extrapolate to VTE prevention strategies in other pediatric cancer diagnoses and children considered at high risk for VTE.

## Introduction

In contrast to the general pediatric population, children with oncologic disorders and thrombosis have an increased risk for death, thromboembolism (TE) recurrence, TE-associated morbidity, and a potential higher utilization of health care resources (1). The reported incidence of venous thromboembolism (VTE) in children ranges from 0.7 to 1.4 VTE/100,000 hospital admissions (2–4). A study using the Nationwide Inpatient Samples (1994–2009) have identified cancer as one of the primary risk factors for pediatric VTE associated admissions (5). A population-based study reported higher absolute VTE risk in children with cancer of 1.52 per 1,000 person-years vs. 0.06 per 1,000 person-years in controls (unaffected children) with a hazard ratio of 28.3 (95% CI 7.0–114.5) (6).

Among all the pediatric malignancies, ALL has the highest cure rate (close to 90%) which has been accomplished through years of collaborative basic science and clinical research (7). Despite achieving this milestone, a large proportion of patients with ALL continue to experience complications related to therapy including VTE. Pediatric ALL has the highest risk for thrombosis compared to other oncologic diagnoses in children. In a large prospective study of 2,183 pediatric patients age ≥12 years old, diagnoses of leukemia and lymphoma were independent risk factors for VTE in children with cancer (8). Thrombosis in ALL is one of the most common therapy-related complications and potentially a preventable one. The reported incidence of thrombosis varies from 1 to 36% (9–17). The majority of thrombosis events are venous and involve the upper central venous system, related to the use and location of central venous catheter (CVC) (18). Most of the thrombosis events typically present during the induction phase of therapy (initial phase) due to the interaction of multiple risk factors such as chemotherapy (e.g., asparaginase, steroids), active disease leading to cell apoptosis contributing to thrombogenicity, and the use of CVC. A meta-analysis by Caruso et al., reported a global risk for thrombosis to be 5.2% in 17 studies of pediatric ALL (95% CI 4.2–6.4) (19). The wide variation in the literature reported incidence of VTE is perhaps related to whether symptomatic or asymptomatic cases were reported, the design of the study (i.e., prospective vs. retrospective) and the chemotherapy regimen used, such as asparaginase during induction therapy or its use during subsequent phases of therapy (induction vs. intensification). The “Prophylactic Antithrombin Replacement in Kids with ALL treated with asparaginase study (PARKAA)” reported a thrombosis prevalence rate of 36.7% (95% CI 24.4–48.8%) in 60 children with ALL and the presence of a CVC during induction therapy (20). These events were largely asymptomatic discovered during the required study radiologic surveillance with only three children (5%, 95% CI 1–14) presenting with symptomatic thrombosis (20). A retrospective study of 1,100 children treated on the Berlin Frankfurt Munster (BFM)-90 trial reported 19 (1.7%) with thrombosis (13). In contrast, a prospective study by Korte et al. reported a higher incidence of thrombosis of 14.3% (11). In summary, depending the study design (e.g., prospective vs. retrospective), the reported overall rates of symptomatic and asymptomatic CVC-related VTE range from 2.6 to 43% (20–22).

Thrombosis events in ALL primarily occur during the induction phase. In the meta-analysis by Caruso et al., 61 TE-events occurred in 1,280 pediatric patients during induction therapy (4.8%, 95% CI 3.7–6.0) while 12 TE-events developed in 609 during later phases of treatment (2.0% 95% CI 1.1–3.3, *p* = 0.004) (19). Regardless of the reported incidence or prevalence of VTE in pediatric ALL studies, thrombosis is associated with acute and long-term sequelae that cannot only impact ALL survival but also place affected children and adolescents to additional long-term complications related to the type and location of the thrombosis event.

## Thrombosis Pathophysiology and Associated Risk Factors in All

Significant plasmatic elevation of the coagulation factors VIII, IX, von Willebrand factor (vWF), and alpha-2-macroglobulin were detected in ALL pediatric patients even before initiation of systemic therapy (23). Other procoagulant proteins such as pre-kallikrein, factor XIII or natural coagulation inhibitors such as protein C were significantly reduced (24). Once systemic chemotherapy is initiated, further hemostatic changes occur, leading to thrombosis and, less commonly, bleeding (25). Chemotherapy can activate platelets and monocyte-macrophage tissue factor, induce cellular apoptosis, and expression/activation of tissue factor leading to a prothrombotic milieu (26–28). The combination of asparaginase and steroids promote a prothrombotic state by an acquired and transient decrease of natural anticoagulants such as antithrombin, protein C and S, resulting in an increase in thrombin generation. Steroids contribute to the pro-coagulable state by increasing the production of procoagulant factors (e.g., XII, XI, IX, X, VIII, VII, V, and II) as well as inhibitors of fibrinolysis such as plasminogen activator inhibitor and anti-plasmin (11, 16). Cytokines and procoagulant molecules produced by cancer cells and endothelial disruption by malignant cells are other contributors to thrombosis risk (29, 30).

Asparaginase is an essential agent for the treatment of ALL, usually incorporated during induction and intensification phases of therapy. Asparaginase antileukemic properties are mediated by asparaginase depletion in leukemic cells, which lacked the enzyme asparaginase synthetase to replenish asparagine. Asparaginase as a result, reduces procoagulant and anticoagulant protein synthesis such as fibrinogen, plasminogen and antithrombin (31, 32). Deficiencies of protein C, S and functional abnormalities of von Willebrand factor have been described as well (10, 23, 33, 34). Therefore, the normal anticoagulant hemostatic function is impaired leading to ineffective regulation of thrombin and this is believed to be the main underlying cause for asparaginase associated thrombosis risk. Several formulations of asparaginase can be grouped into short-acting (e.g., Erwinase and *E. coli* asparaginase) and long-acting (e.g., PEG-aspargase). Some reports showed a less pronounced effect on coagulation factor levels with Erwinase compared to *E. coli* asparaginase (32, 35–38). In the meta-analysis by Caruso et al., exposure duration to asparaginase correlates with thrombosis risk. There was more thrombosis in patients receiving ≤ 6,000 U/m^2^ over long exposure times compared to ≥10,000 U/m^2^ for short exposure time (19).

Steroids are another essential drug in the treatment of leukemia. Prednisolone induces elevation of factors VIII, vWF, prothrombin, and antithrombin levels (39–42). In addition, it favors a “hypofibrinolytic state” with an increase in plasminogen activator inhibitor-1 levels (40, 43). The combination of prednisolone and asparaginase levels increases vWF-antigen and its large molecular weight multimers (44). The BFM and COALL ALL study groups reported a prospective study of 420 patients (BFM *n* = 300, COALL *n* = 120), that confirmed the higher thrombosis incidence with concomitant administration of asparaginase and steroids (BFM 11.6%, COALL 2.5%) (OR 7.7 *p* = 0.05) (16). In the BFM studies, asparaginase is administered during induction with steroids, whereas in COALL, asparaginase is given during consolidation without concurrent steroids. The type of steroid used also seems to change thrombosis risk. A historical comparison of two BFM studies showed a reduced thrombosis risk with the use of dexamethasone compared to prednisolone (BFM 2000-Dexamethasone = 1.8%; BFM 90/95 prednisolone = 10.4% *p* = 0.028) (45). In contrast to the previous study, in a meta-analysis of ALL studies, there was no statistical difference in thrombosis incidence according to the type corticosteroids used. Still, prednisolone was associated with a higher TE risk following induction treatment (12.2% vs. 1.6% *p* = 0.001) (19).

Age has been reported as a risk factor for thrombosis. Data from DFCI 91-01 showed older children (ages ≥ 9 y) had a higher incidence of thrombosis compared to the younger age group (15% vs. 2%; *p* < 0.01) (17). Athale et al., similarly reported older age as a risk factor for thrombosis in the DCFI-ALL 95-01 and 2001-01 (46). In a prospective Nordic-Baltic study for ALL in children and adults (ages 1–45 y) treated on the NOPHO ALL 2008 (2008–2017), the 2.5-year cumulative incidence of VTE was 7.9% (95% CI 6.6–9.1) higher in patients aged at least 10 years of age (*p* = 0.0001) (47). Adjusted hazard ratio (HRa) for ages 10–17.9 y was 4.9 (95% CI 3.1–7.8 *p* < 0.0001) and for ages 18–45.9, 6.06 (95% CI 3.65–10.1 *p* < 0.0001). At diagnosis, patients with a mediastinal mass had a HRa of 2.1 (95% CI 1.0–4.3; *p* = 0.04). In a multiple absolute risk regression model, age at least 10 years had the largest absolute risk ratio (RR 4.7; 95% CI 3.1–7.1), enlarged lymph nodes RR 2.0 (95% CI 1.2–3.1), and mediastinal mass RR 1.6 (95% CI 1.0–2.6) (47). An increased hazard for pulmonary embolism was found in patients older than 18 y (HRa 1.6; 95% CI 4.02–33.7 *p* < 0.0001). Cerebral sinus venous thrombosis had an elevated hazard rates for children and adolescents 10–17.9 y (HRa 3.3; 95% CI 1.5–7.3 *p* = 0.003) compared to children younger than 10 y (47). Five deaths were secondary to VTE and children younger than 18 y who had developed a VTE had an increased hazard of dying compared to the same patients without VTE (*p* ≤ 0.01) (47). However, age by itself might not be the only biological risk factor for the associated greater risk for thromboembolic complications, but rather the delivery of more intensive therapy recommended for older children, which are considered high-risk ALL due to age (46).

Among all pediatric malignancies, children with ALL are more likely to develop CVC-associated thrombosis (14, 48). The majority are asymptomatic and most frequently at the CVC venous anatomic location (19, 49). Central venous catheter-associated thrombosis can result in acute and long-term complications, including interruption of therapy, thrombosis recurrence (4–19%), post-thrombotic syndrome (5–25%), pulmonary embolism (8–15%), and death (21, 50). In a meta-analysis of 11 studies, peripherally inserted central catheters were associated with a 2.5-fold higher risk for DVT than centrally inserted venous catheters or surgically placed CVC (51). External CVC were found to be associated with a higher risk for DVT compared to internally placed CVC in another report (52). In a PARKAA sub-study, the risk for CVC related thrombosis was higher with right-sided catheters (OR 2.5, 95% CI 1.0–6.4 *p* = 0.048), subclavian insertion (OR 3.1, 95% CI 1.2–8.5 *p* = 0.025), and those placed percutaneously (OR 3.5, 95% CI 1.3–9.2, p-0.011) (49). In a retrospective study of the Pediatric Oncology Group 9201, externalized CVC was associated with a higher thrombosis risk when compared to the fully implanted CVC (OR 3.9, 95% CI 1.5–10.3, *p* = 0.006) (52). Central venous catheter-associated thrombosis was retrospectively reviewed in a cohort of 114 oncology patients (median age 6.04 years old) of which 66 (58%) were hematopoietic tumors (*n* = 38 ALL) and the remaining solid tumors. At the time of magnetic resonance venogram, 42 children (37%) had their CVC in place, and 72 (63%) had the device removed. CVC associated thrombosis occurred 45 (39.5%) children of which 14 (31%) had ALL. Younger age at diagnosis, female sex, duration of CVC, left sided placement of the CVC were independently associated with thrombosis. Their findings of younger age as a risk factor for thrombosis might be related to the inclusion of solid tumors in their analysis. Asparaginase therapy and prothrombotic risk factors were not. Mild PTS characterized by the presence of collateral vessels was present in 5.6% of children (53).

Inherited thrombophilia increases the risk for thrombosis primarily through the interaction of environmental and individual host factors. It is not clear how screening for thrombophilia will help develop preventive strategies for thrombosis prevention in ALL. Several studies have found an increased risk for thrombosis in children with ALL with inherited thrombophilia. In a multicenter BFM 90/95, the risk for thrombosis was higher in those with a thrombophilia defect (46.5 vs. 2.2% *p* < 0.0001), and those with multiple defects had a much higher thrombosis risk (*p* = 0.009) (54). Among 82 children with ALL treated according to the BFM 95 protocol, thrombosis occurred in 10 (12%) of the patients. Thrombosis risk was found to be significantly higher in patients with factor V Leiden (FVL) (OR = 7.1; 95% CI 1.6–30.5). Thrombosis frequency in children 10 years or older was significantly higher than those younger than 10 years of age (70 vs. 30%, *p* = 0.03). No correlation with thrombosis risk was found among those patients with FVL and prothrombin 20210A genetic mutations in the PARKAA study; however the study lacked the power to detect a meaningful difference with congenital thrombophilia (20). The meta-analysis by Caruso et al., showed that inherited thrombophilia increased the risk for thrombosis by 8-fold (19). The combined effect of congenital thrombophilia and therapy with *E. coli* asparaginase and prednisolone increased the risk of thrombosis (OR 34.5 95% CI 4.39–271.42, *p* = 0.0008) (16). While there is a debate about the presence of thrombophilia and its role in cancer-associated VTE, some experts recommend that assessment of thrombophilic risk factors can assist in the decision whether to consider the longer duration of anticoagulation therapy in VTE or anticoagulant prophylaxis in high-risk malignancies (55).

**Figure 1:**
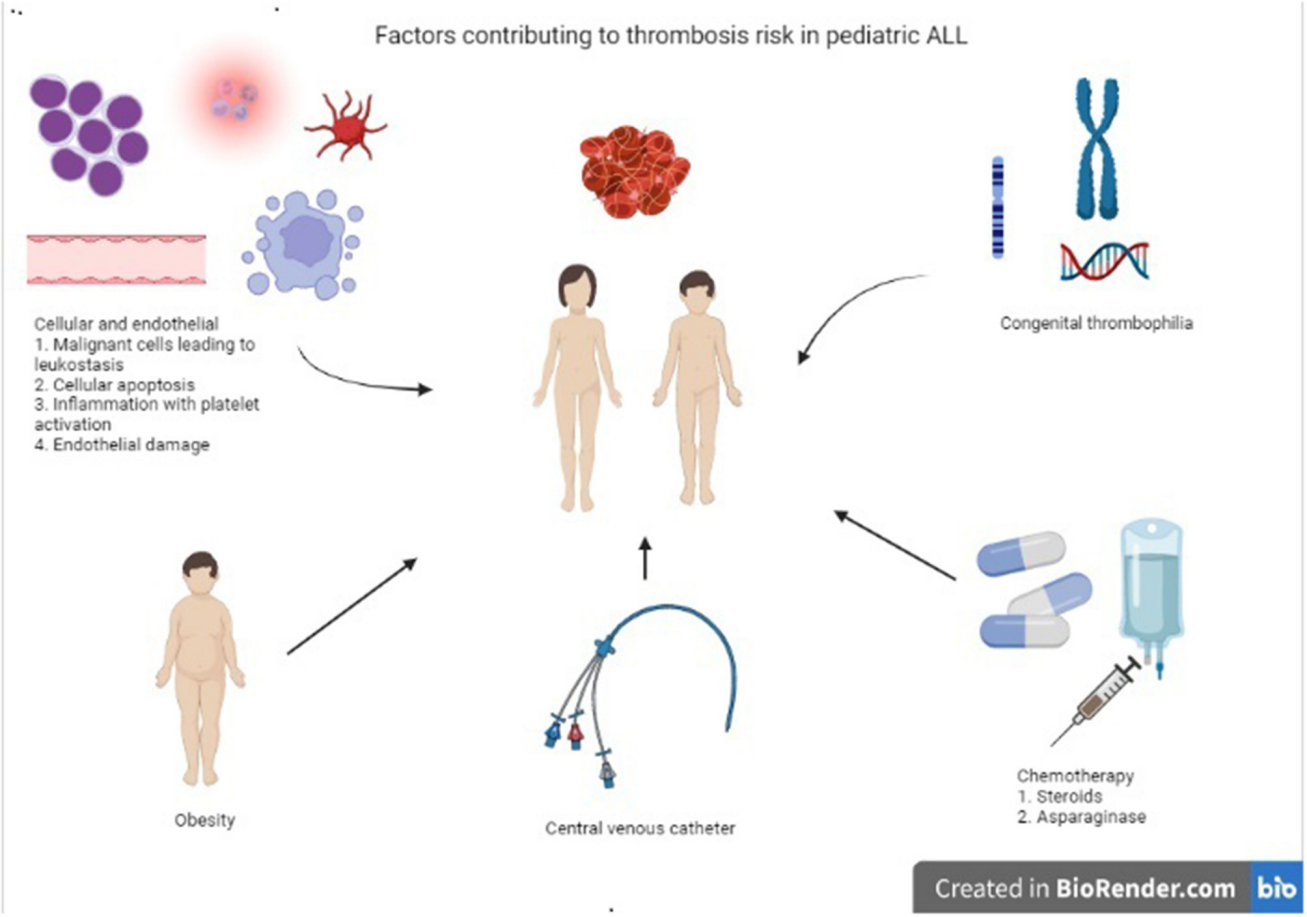
Summary most common risk factors contributing to thrombosis risk in children, adolescents, and young adults with ALL.

Obesity, defined as a body mass index ≥30 kg/m^2^, is another biologic risk factor frequently identified as a risk factor for thrombosis. Between 1980 and 2012, obesity has doubled in children aged 6–11 years (7 to 18%) and quadrupled in adolescents aged 12–19 (5 to 21%) (56). Several hemostatic alterations have been observed in obese individuals that can explain the thrombosis elevated risk such as altered fibrinolysis, increased plasminogen activator inhibitor (PAI-1), increased platelet aggregability, through elevations of von Willebrand factor, protein C resistance and higher levels of fibrinogen, factors VII and VIII (57, 58). In a retrospective study of children with ALL treated during 2008–2016 (*n* = 294), 9.2% developed symptomatic VTE (*n* = 27), with 70% occurring during induction. Obesity was strongly predictive of symptomatic VTE with an OR 3.8 (95% CI 1.5–9.6, *p* = 0.008) (59). As obesity continues to rise in our pediatric population, it will need to be considered as an additional thrombosis risk factor, especially in those diagnosed with ALL. Figure 1 summarizes the previously discussed thrombosis risk factors in ALL.

## Thrombosis Anatomic Sites in All

Thrombosis events are largely venous in pediatric ALL. A review of symptomatic cases on children with ALL on pediatric trials from 1966 to 2003 found 73% to be venous events (28% central nervous system (CNS) and 45% non-CNS) (1). Venous thrombosis events are significantly related to the presence of a CVC with a reported incidence of 30–50% (1, 49).

## Central Venous Catheter Associated Thrombosis

Please refer to Thrombosis pathophysiology and associated risk factors in ALL section.

## Central Nervous System Events

The cerebral sinus venous thrombosis (CSVT) incidence, prevalence, the timing of the event, associated risk factors, neurologic morbidity, and mortality varies among the different ALL clinical studies. The Dutch Childhood Oncology Group (DCOG) ALL-10 (2004–2013) treated 778 children with ALL (T or B cell) ages 1–18 y with a reported 59 VTE (7.6%), 26 of the thrombosis events being CSVT (44.1%). Over half (59.3%) occurred during induction therapy, and the remaining occurred during medium risk intensification. Conditional multivariate logistic regression analysis showed age and ALL subtype to be significantly associated with VTE occurrence (age ≥ 7 y; OR 2.72; 95% CI 1.33–5.57; T cell ALL subtype OR 2.95 (95% CI 1.02–8.57). A multivariate Cox model showed no association between the occurrence of VTE and EFS. Close to a third of the patients (34.6%) with CSVT, neurologic disability was present (60). Among 346 patients ages 1–16 y treated according to DFCI study from 1998 to 2011, central nervous system (CNS) thrombosis occurred in 3.8% (13 of 346) (95% CI 2.0–6.3%). Twelve were diagnosed during intensification, all resolved within 2 weeks without neurologic sequelae or survival impact. Obesity, defined as a body mass index > 95^th^ percentile, was identified as a thrombosis risk factor with an OR = 3.37 *p* = 0.064. Patients receiving *Erwinia asparaginase* had a significant risk reduction for CSVT occurrence (OR = 0.12 *p* = 0.018) (61). The UKALL 2003 trial reported 46 CSVT among 3,126 participants ages 1–24 during 2003–2011 with an incidence of 1.4%. The vast majority occurred during induction (77%), all receiving asparaginase, at a median time from leukemia diagnosis to thrombosis of 29 days (IQR 22–35). Four patients with CSVT died, one due to a CNS event and intracranial hemorrhage (62). Neurological morbidity was reported in five cases (12%) at a median follow-up of 41 months. The Nordic Society of Pediatric Hematology-Oncology (NOPHO-ALL2008) reported CSVT cumulative incidence of 2% (20 of 1,038 children): 16 related to asparaginase and 16 related to steroids. Most of the events occurred during consolidation. Nearly all patients were treated with LMWH without bleeding complications. The mortality related or indirectly related to CSVT was 10% (63). A meta-analysis of 17 prospective studies comprising 1,752 children with ALL estimated the rate of CNS thrombosis including cerebral infarction and stroke to be 2.9% (95%CI 2.2–3.8) and for non-CNS 2.3% (95%CI 1.7–3.2) (19). A study from the UK reported a CSVT incidence of 1.1% among 1,824 children with ALL (64). In an American study that included children with cancer and stroke, two had CSVT among 190 children with ALL (1.1%) (65). Risks factors for CSVT were studied in a cohort of 209 patients ages 1–21 with ALL (66). The incidence of CSVT was 6.2% (95% CI 3.4–10.4). Univariate wanalysis showed that age >10 years (OR 3.56; 95% CI 1.13–11.2), T-cell immunophenotype (OR 4.14; 95% 1.16–14.7) and intermediate/high risk disease (OR 3.4; 95% 1.03–11.7). The only statistically significant risk factor by multivariate analysis was treatment per intermediate/high-risk protocol (HR 15.6; 95% CI: 1.43–111.3). Seventy-seven percent of the cases occurred post-induction phase while receiving asparaginase and dexamethasone rather than prednisone. Patients were treated with LMWH for a minimum of 3 months and had 100% survival rate. All but one patient had neurologic recovery (66).

## Cardiac and Arterial Events

Less commonly reported sites for thrombosis are intracardiac and arterial thrombosis events (e.g., stroke and pulmonary embolism). Right atrial thrombosis has been reported more frequently in those studies screening for asymptomatic thrombosis (1, 48). Korones et al., reported a prevalence of right atrial thrombosis in children with cancer and a CVC of 8.8%, whereas in the PARKAA study it was 13.6% (45, 48). Arterial thromboses are less frequently reported in contrast to venous thrombosis events. In a retrospective study of arterial stroke among children with ALL, 11 symptomatic strokes occurred among 2,318 patients (0.47%) (67). In a smaller cohort study of 253 children with ALL, five children experienced a stroke (3 hemorrhagic and two ischemic) during asparaginase therapy (68).

## Pulmonary Embolism

Pulmonary embolism (PE) can be a life-threatening complication during ALL therapy. The NOPHO ALL 2008 study reported PE events in 32 of 1,685 patients enrolled in their trial (69). The 2.5-year cumulative incidence of first-time PE increased with the age groups: 0.43% (95% CI, 0.18–1.03) in children aged 1–9 years, 3.28% (95% CI, 1.72–6.22) in children aged 10–17 years, and 7.22% (95% CI, 4.61–11.21) in adults aged 18–45 years. Seventy-eight percent of the PE events (25/32) occurred during asparaginase treatment. In two-thirds of the patients (63%; 17/27), PE and its treatment had no impact on the cumulative prescribed doses of asparaginase. Their study reported a PE-associated 30-day mortality of 9.4% (95% CI, 1.9–25.0) (69). Predictive models to assist in the diagnosis of PE need to be further studied in pediatric and young adults with ALL.

## Thrombosis: Acute and Long-Term Sequelae

Most of the thrombotic events and their related outcomes have been studied and reported primarily in symptomatic thrombosis. Post-thrombotic syndrome (PTS) is a chronic complication following deep vein thrombosis (DVT) characterized by signs and symptoms of venous insufficiency such as limb edema, pain, and skin ulceration in more severe cases (70). Reported pediatric PTS rates range from 3 to 70% in those children who were followed for 2 years following the thrombosis event (71, 72). In the adult population, PTS rates have been reported approximately 17% at 1 year and 60% by 2 years (73, 74). Post-thrombotic syndrome have been described in 30–35% of children with a history of cancer after CVC discontinuation or removal (75).

Although the clinical significance of asymptomatic thrombosis has been debated in the pediatric thrombosis literature, these events might also lead to long-term sequelae. In a smaller report, 14 of 16 patients (88%) with asymptomatic thrombosis had two or more catheter infections and or occlusions before the thrombosis diagnosis (76). Nearly half of childhood ALL survivors who were diagnosed with asymptomatic thrombosis have PTS, which is a similar PTS prevalence to those patients with symptomatic VTE (71, 77). Kuhle et al., studied PTS in 13 children with asymptomatic thrombosis in the PARKAA study. Seven patients (54%; 95% CI: 25–81%) showed signs and symptoms of PTS. All patients demonstrated collateral circulation on exam and three had increased arm circumference. Follow-up venous Doppler ultrasonography evaluation in six of the 13 patients demonstrated thrombosis with collaterals. One patient complained of aching pain while writing (77). In a cross-sectional study assessing PTS in ALL survivors without DVT, after a mean follow up of 9.5 years, 10 of 41 (24%; 95% CI: 11–38%) had signs of PTS, including collateral veins in 10 patients (100%) and increased arm circumference in five (50%) (77).

Conflicting data exists whether the development of cancer associated VTE has a negative impact on survival. In a population-based retrospective cohort study using the “Cancer in Young People Canada Registry”, 2006 children < 15 years of age (median age 4 years) with the diagnosis of ALL (88.5% B-cell precursor ALL) were studied for VTE and survival outcomes (78). Thromboembolism occurred in 113 patients (5.6%) at a median time of 107 days (IQR 35–184) from ALL diagnoses. Among the standard risk ALL group, VTE was diagnosed in 3.5% (41/1,165) vs. 8.6% (72/841) in the high risk ALL group. Patients with VTE had worse overall survival with a HRa for death of 2.61 (95% CI 1.62–4.22) *p* < 0.001) and EFS (HRa of death, relapse, second malignancy: 2.03 95% CI 1.35–3.05 *p* = 0.001) compared to patients without VTE. Thromboembolism was associated with significantly lower overall survival and event-free survival in children with high/very high-risk ALL (HRa 2.90, 95% CI 1.79–4.72, *p* < 0.001 and HRa 2.02, 95% CI 1.30–3.12 *p* = 0.002, respectively) (78). Omission of asparaginase can result in inferior leukemic survival. In the DFCI, patients receiving < 25 weeks of asparaginase therapy due to different adverse events, including thromboembolism, their EFS was lower than those receiving ≥ 26 weeks (73 vs. 90% *p* < 0.01) (17). Similar to the DFCI group findings, the Children's Oncology Group reported an inferior disease free survival for those patients with high risk ALL not receiving the prescribed asparaginase doses (HR 1.5; 95% CI 1.2–1.9; *p* = 0.002) (79). In a report of children and adults (*n* = 548) treated with the DFCI regimen, VTE occurred in 43 patients (8%), 5% were pediatric, and 34% were adults. Complications from VTE included post-thrombotic syndrome (7%), neurological changes (headaches, seizures 20%), catheter removal (21%), and discontinuation of asparaginase administration (14%) (80). Adults had a higher rate of PTS compared to children (*p* = 0.05); all other complications were similar among the two age categories. There was no difference in EFS and OS when comparing those with and without VTE. Thirty patients (70%) received at least 85% of the prescribed asparaginase. About a third of these patients with thrombosis (33%) developed recurrent VTE. Recurrent VTE was more frequent in adults compared with the pediatric patient population treated in this trial (pediatric 17% vs. adults 47% *p* = 0.07) (80).

Long-term neurologic outcomes in the general pediatric population with CVST have identified cognitive difficulties, attention, and perception problems and epilepsy as complications associated with CNS thrombosis (81, 82). Ross et al., reported on long-term neurological outcomes in children with ALL/Lymphoblastic lymphoma and CSVT treated at a single institution. With a median follow-up of 8 years (range 5–10), nine children among 200 developed CSVT (83). Four of these children and adolescents completed neurologic evaluation. Neurological examination was normal, but their neuropsychological testing showed “average to above average function on cognitive and behavioral testing”. Three patients had problems with visual-motor integration, and one patient had difficulties with fine-motor dexterity and non-verbal memory. None of the patients were aware of these neurological deficits (83). More extensive studies in pediatric ALL patients with CSVT are needed to determine the neurocognitive impact of this complication.

## Thrombosis Treatment and Prevention in All

Low molecular weight heparin (LMWH) is considered the drug of choice and the most commonly agent utilized to treat thrombosis in ALL due to several pharmacological properties, including lack of drug or diet interactions and the ability to reverse anticoagulation effects before invasive procedures such as lumbar punctures, by holding the medication administration for at least 24 hr. The American College of Chest Physicians recommends at least 3 months of anticoagulation therapy for DVT associated with cancer and extended treatment if no thrombosis resolution (84). Prophylaxis with LMWH should continue until all risk factors are no longer present, such as the presence of a CVC. If CVC associated thrombosis and the CVC is still functional, it is recommended to keep the CVC if needed for the continuation of systemic chemotherapy (84).

In adults with cancer at higher risk for thrombosis, the recommendation is to use prophylactic anticoagulation upon initiation of therapy (85, 86). Novel anticoagulants such as direct oral anticoagulants (DOACs) had been studied in adults with cancer, showing non-inferiority compared to standard of care-SOC (LMWH) for the composite outcome of first recurrent VTE or major bleeding (87). A recent meta-analysis on the use of DOACs in adult cancer patients concluded that its use is more effective in preventing recurrent VTE when compared to LMWH however, they convey a higher bleeding risk (88). So far, prophylactic anticoagulation in pediatric ALL is not considered the SOC. Recent studies have explored the role of prophylaxis in preventing VTE during ALL therapy, primarily in the phases of treatment considered higher risk for thrombosis, such as induction therapy (Table 1).

**Table 1:** Summary of VTE prevention in pediatric ALL and other oncologic diagnoses.

**Study**	**Population**	**Intervention**	**Outcome**
Hongo et al. (89)	• Japan Association of Childhood Leukemia Study (JACLS) ALL-97 protocol (*n* = 127) • Multicenter retrospective survey	• Forty-eight patients (38%) received AT III concentrate • A greater percentage of patients in the AT III-supplement group received FFP	• Clinically relevant hemostatic complications: (1.6%; 2/127), one ICH, one stroke • Prophylactic administration of AT III concentrate or FFP: no difference in VTE prevention
Mitchell et al. (20)	• Pediatric randomized controlled AT replacement in children with ALL undergoing asp therapy induction therapy	• AT replacement if AT <30 U/L. • Radiological imaging used: bilateral venography, ultrasound venous Doppler, echocardiogram, and MRI	• VTE was present in 7 of 25 patients treated with AT (28%; 95% CI 12.1–49.4%) vs. 22 of 60 patients (36.7%; 95% CI 24.4–48.8%)
Ruud et al. (90)	• Randomized, controlled study for the prevention of CVC-associated VTE in children with newly diagnosed malignancies and CVC placed in the jugular vein	• Low dose warfarin with an INR goal of 1.3–1.9 in 31 patients compared to a standard of care control 42 patients	• CVC associated thrombosis was no different among the study arms (VTE warfarin arm 48% vs. 36% in the control arm).
Meister et al. (91)	• Prospective study combined LMWH prophylaxis (1 mg/kg/day) and AT supplementation vs. AT alone (with a non-contemporaneous control). • 112 children with newly diagnosed ALL treated according to the BFM 95/2000 protocols with the use of *E. coli* asp	• AT levels > 50% with replacement therapy during induction therapy.	• Symptomatic VTE developed in 12.7% of the children that did receive AT prophylaxis (*n* = 71; 95% CI 6.0–22.7) vs. no VTE with LMWH/AT prophylaxis (*n* = 41, 95% CI 0.0–8.6 *p* <0.05).
Sibai et al. (85)	• Single center retrospective cohort study of Philadelphia-negative ALL receiving asparaginase based intensification • 125 children (2011–2017) compared to a historical control (*n* = 99)	• Pharmacologic prophylaxis with LMWH	• 17 children developed VTE (13.6%) pharmacologic prophylaxis group while 27 VTE events occurred (27.3%) in the control arm (OR 0.42 95% CI 0.21–0.83).
Greiner et al. (92)	• THROMBOTECT Prospective randomized trial comparing efficacy and safety of antithrombotic therapy in the leukemia trials ALL BFM 2000 and AIEOP-BFM ALL 2009. • Patients with newly diagnosed ALL ages 1–18 y (*n* = 949)	• Patients randomized to receive low dose unfractionated heparin (2 U/kg/hr), prophylactic LMWH (enoxaparin) or AT replacement during induction therapy	• VTE 42 patients (4.4%). • Unfractionated heparin group had higher risk TE (8.0%) compared with those randomized to enoxaparin (3.5% *p* = 0.011) or AT (1.9% *p* <0.001). • Major hemorrhage occurred in eight patients without differences among treatment arms. • 5-year EFS 80.7 ± 2.2% AT arm compared to 85.9 ± 2.0% in the enoxaparin group (*p* = 0.10).
O'Brien et al. (93)	• PREVAPIX-ALL Open label randomized controlled trial in VTE prevention in children with ALL and LLy and the presence of a CVC, using a DOAC (apixaban) during asparaginase containing induction therapy. • Ages 1–18 years old treated with PEG-asparaginase during induction therapy and presence of a CVC	• Apixaban prophylactic doses compared to SOC	• Study completed randomization and accrual-awaiting publication of results

*AT, antithrombin; VTE, venous thromboembolism; Asp, asparaginase; DOAC, direct oral anticoagulant; MRI, magnetic resonance imaging; FFP, Fresh frozen plasma; ICH, intracranial hemorrhage; SOC, standard of care*.

Antithrombin (AT) is the primary plasmatic anticoagulant transiently decreased secondary to asparaginase. Fresh Frozen Plasma (FFP) and AT replacement have been used to correct AT deficiency temporarily. Most of the studies using FFP supplementation during asparaginase therapy did not show a significant increase in AT levels (89, 94, 95). Fresh frozen plasma has the potential to antagonize the effects of asparaginase by replacing asparagine. Hongo et al., reported the use of AT replacement in 48 of 127 patients (38%) (96). AT levels were lower than the non-replacement group, and no differences in VTE events were observed among the two groups although the study sample size and thrombosis events were small. Nowak-Gottl et al., reported the use of AT concentrate in 15 of 27 patients treated according to ALL-BFM-90 protocol. Replacement therapy was completed once AT levels decreased to 60% or less. The use of AT “normalized thrombin generation and decreased d-dimers and plasminogen activator inhibitor” (36). The PARKAA study is the largest pediatric randomized controlled AT replacement clinical trial in children with ALL undergoing asparaginase therapy. Eighty-five children with ALL participated in this clinical study. For the therapy arm, AT was replaced once levels were <30 U/L. Thrombosis was diagnosed by use of bilateral venography, ultrasound venous Doppler, echocardiogram examination, and magnetic resonance imaging. VTE was present in seven of 25 patients treated with AT (28%; 95% CI 12.1–49.4%) vs. 22 of 60 patients (36.7%; 95% CI 24.4–48.8%) (20).

Several studies had explored the pharmacologic prophylaxis role in thrombosis prevention in children, primarily those with CVC. The PROTEKT study (“Prophylactic LMWH compared with SOC for the prevention of CVL-related VTE”; this study included non-oncologic diagnoses as well), was an open-label, multi-center randomized study using reviparin for the prevention of CVL associated VTE. Seventy-eight patients were treated with reviparin and 80 as per SOC. No major bleeding was encountered in the reviparin arm. The study had to be closed early due to slow accrual, with only 31% of the required number of patients enrolled in the study. Due to early study closure, the study was underpowered to demonstrate either the safety or efficacy of pharmacologic prophylaxis (90).

A randomized, controlled study for the prevention of CVC-associated VTE in children with newly diagnosed malignancies and CVC placed in the jugular vein used low dose warfarin with an INR goal of 1.3–1.9 in 31 patients and a standard of care control in 42 patients (91). CVC associated thrombosis was no different among the study arms. The study was terminated early (VTE warfarin arm 48 vs. 36% in the control arm) (91). Only a few patients achieved the INR goal pre-defined by the study illustrating the challenge of anticoagulation with warfarin in the pediatric population.

A prospective study was conducted to determine the influence of combined LMWH prophylaxis (1 mg/kg/day) and AT supplementation vs. AT alone (with a non-contemporaneous control), on the incidence of VTE in 112 children with newly diagnosed ALL treated with *E. coli* asparaginase according to the BFM 95/2000 protocols (97). Antithrombin plasma levels were maintained above 50% with AT replacement therapy during induction therapy. Symptomatic VTE developed in 12.7% of the children that did receive AT prophylaxis (*n* = 71; 95% CI 6.0–22.7) compared to no VTE in children receiving combined prophylaxis (*n* = 41, 95% CI 0.0–8.6 *p* < 0.05). Thrombosis events were: CSVT = 3, lower extremity DVT = 3, upper extremity DVT = 2, and upper DVT combined with pulmonary embolism (*n* = 1) (97). A non-randomized ALL cohort study studied prophylactic enoxaparin during L-asparaginase therapy in VTE prevention in 41 children compared to a historical control (*n* = 50) (92). No symptomatic VTE or bleeding complications occurred in the enoxaparin arm. Thrombosis complications occurred in the historical control arm (1 DVT and 1 PE).

A single-center retrospective cohort study of Philadelphia-negative ALL receiving asparaginase based intensification therapy studied the role of pharmacologic prophylaxis in 125 children (2011–2017) compared to a historical control (*n* = 99) who received treatment during 2001–2017 (86). Seventeen children developed VTE (13.6%) therapy in the pharmacologic prophylaxis group, while 27 VTE events occurred (27.3%) in the control arm (OR 0.42 95% CI 0.21–0.83). The VTE sites were lower extremity DVT 54.6%, PE 13.6%, and CVC-related thrombosis 22.7%. No major bleeding occurred.

The “THROMBOTECT study” was a large prospective randomized trial that compared the antithrombotic therapy safety and efficacy in the leukemia trials ALL BFM 2000 and AIEOP-BFM ALL 2009. Patients with newly diagnosed ALL ages 1–18 y (*n* = 949) were randomized to receive low dose unfractionated heparin (2 U/kg/hr), prophylactic LMWH (enoxaparin) or AT replacement during induction therapy (98). Thromboembolism occurred in 42 patients (4.4%). Those assigned to receive unfractionated heparin had higher risk TE (8.0%) compared with those randomized to enoxaparin (3.5% *p* = 0.011) or AT (1.9% *p* < 0.001). A large proportion of patients refused allocation to randomized treatment arms: 3% low dose heparin and AT replacement therapy and 33% in the enoxaparin arm. Major hemorrhage occurred in eight patients without differences among treatment arms. The 5-year EFS was 80.7 ± 2.2% among patients assigned to AT arm compared to 85.9 ± 2.0% in the enoxaparin group (*p* = 0.10). Prophylactic use of AT or enoxaparin significantly reduced TE. It is unknown how AT replacement might have affected leukemia EFS in this study (98).

A systematic literature review and meta-analysis study of primary pharmacological (e.g., LMWH, VKA, AT) prophylaxis in children and adolescents with cancer (0–21 years) compared to SOC was conducted, to determine the different pharmacologic agents role in VTE prevention (99). Six articles describing 1,318 patients were included (mean age 6.7 years, 56.7% males), with ALL comprising 97.5% of patient diagnoses. All studies were considered moderate or high risk of bias. Low molecular weight heparin was the only agent found to be associated with lower odds of TE compared to SOC (OR 0.23, 95% CI 0.06–0.81). No statistical differences were detected between thromboprophylaxis modalities, and SOC. No difference in the odds of major bleeding was found among the different treatment arms (99).

More pediatric clinical trials studying the safety and efficacy of direct oral anticoagulants (DOACs) for the treatment of DVT in children have been recently published, showing that this type of novel oral anticoagulant agents is safe and non-inferior when compared to LMWH (100–102). In the Einstein Jr phase three clinical trial comparing rivaroxaban to SOC in children with VTE, 10–12% of the pediatric patient cohort had the cancer diagnosis (100). Similarly, to the Einstein Jr study, 11% of children enrolled in the Dabigatran etexilate Diversity studies had the primary diagnosis of hematological malignancies (101, 102).

A recently published systematic review of DOACs use in 1,007 in children treated in phase I to phase IV, reported rivaroxaban and dabigatran to be as effective as traditional anticoagulants with a low frequency of recurrent thrombosis and bleeding rates (93). The “PREVAPIX-ALL Study [apixaban compared to SOC for the prevention of venous thrombosis in pediatric ALL and lymphoblastic lymphoma (LLy)]” is the first open label randomized controlled trial in VTE prevention in children with ALL and LLy using a DOAC (apixaban) during asparaginase containing induction therapy compared to no anticoagulation SOC. The primary efficacy endpoint of the study was a composite of symptomatic and asymptomatic VTE (DVT, PE, CSVT, and VTE-related death) with bleeding as the primary safety outcome (103). The study has recently completed accrual, and analysis of the data is underway.

## Discussion (and Future Directions)

Pediatric ALL remains one of the most curable oncologic diagnoses in children and adolescents. A more disease risk-stratified intensity of therapy has achieved these favorable cure rates; however, chemotherapy is often associated with acute and long-term complications, with VTE being one of the most common and potentially preventable. Thrombosis-related complications can result in acute morbidity leading to interruption or omission of therapy, which can negatively impact disease prognosis. It can also lead to thrombosis-associated mortality and potential long-term complications depending on the anatomic site and extent of the thrombosis event (e.g., PTS in extremity-associated thrombosis, neurologic complications in CSVT). Anticoagulant therapy in pediatric ALL thrombosis has shown to be safe with low risk for major bleeding complications. Different preventive therapy approaches have been studied, including AT replacement, anticoagulation with LMWH or warfarin, and most recently the use of DOAC prophylaxis during induction chemotherapy incorporating steroids and asparaginase (Table 1). More studies looking at thrombosis-related quality of life measures and the impact of VTE preventive strategies are needed.

Several questions remained to be answered: is pharmacologic prophylaxis recommended for all ALL patients? Is there is a particular group considered at higher risk for VTE? For those considered at higher risk for VTE, is prophylaxis indicated past induction phase? If prophylaxis is initiated, what is the recommended time window? Before or after CVC placement? Does prophylaxis prevent CVC loss due to occlusion? Infection? Pharmacological prophylaxis therapy needs to be tailored according to the different VTE risks associated with the different ALL therapy protocols and how asparaginase and steroids are incorporated into the treatment backbone. In addition, genetic thrombophilia risk factors are more prevalent in some ethnic groups (e.g., Factor V Leiden in Caucasian individuals), which might contribute to their thrombosis risk however, they might not be applicable to specific ALL patient populations, whereas congenital thrombophilia prevalence is low.

Predictive VTE risk models might be a helpful tool to help direct which patient population might benefit from prophylaxis. Mitchell et al., developed and validated a predictive VTE model (104). The risk for VTE was low if ≤ 2.5 or high if ≥ 2.5. The model was validated in children treated according to BFM, COALL, and FRALLE protocols. Children with a high VTE risk score had a significantly reduced thrombosis-free survival when compared to children with low score. VTE developed in eight of 322 children (2.5%) with low scores and 11 of 17 (65%) with high scores (104). In a pilot study, eight of 19 children with ALL and high-VTE risk using Mitchell et al., predictive VTE model, pharmacologic prophylaxis was given before CVC insertion. The children receiving LMWH showed a better thrombosis free survival compared to those who did not receive pharmacologic prophylaxis with LMWH (*p* = 0.023) (104). Al-Aridi et al., showed that the predictive model developed by Mitchell et al., might not apply to all ALL-treatment protocols. Eight of 11 children treated according to the St Jude XV protocol developed VTE, with six of eight occurring outside the induction phase, felt to be the concurrent dexamethasone and asparaginase used during the intensification phase. None of the patients in this therapy regimen would have been eligible for pharmacologic prophylaxis based on the Mitchell et al., VTE prediction model (105).

These are exciting times for those searching for an anticoagulant that is “more patient-friendly” in terms of route of administration while at the same time, safe to use in pediatric VTE prevention. Children and adolescent cancer survivors have a longer life expectancy compared to adults, and as a result, will suffer from long-term complications related to cancer therapy, including thrombosis. The introduction of DOACs has opened research opportunities to study these agents in the treatment and secondary prevention of VTE in pediatric ALL and primary prevention those children considered at higher risk for DVT, such as those who required a long-term CVC for their care. The most recent DOAC studies have demonstrated non-inferiority in terms of efficacy and safety when compared to standard anticoagulation (LMWH) in pediatric thrombosis. The results of the PREVAPIX study will shed more light on the safety and activity of thrombosis prevention with the use of DOAC in pediatric ALL. Future studies will provide further evidence of the safety and effectiveness of using these agents in selected higher VTE risk patients with ALL and other oncologic diagnoses.

## Author Contributions

VR is the only author who designed the concept, reviewed the literature, and discussed potential future research in this topic of thrombosis prevention in ALL.

## Conflict of Interest

The author declares that the research was conducted in the absence of any commercial or financial relationships that could be construed as a potential conflict of interest.

## Publisher's Note

All claims expressed in this article are solely those of the authors and do not necessarily represent those of their affiliated organizations, or those of the publisher, the editors and the reviewers. Any product that may be evaluated in this article, or claim that may be made by its manufacturer, is not guaranteed or endorsed by the publisher.
